# Ruthenium(II) Carbosilane Dendrimers With Aliphatic Polyamine Ligands for In Vivo Antimelanoma Activity

**DOI:** 10.1155/bca/4574339

**Published:** 2026-10-26

**Authors:** Yoel Garrosa-Miró, Lorena García Hevia, Mónica L. Fanarraga, Paula Ortega, Francisco Javier de la Mata

**Affiliations:** ^1^ Department of Organic Chemistry and Inorganic Chemistry Research Institute of Chemistry ‘Andrés M. Del Rio (IQAR)’ Institute “Ramón y Cajal” for Health Research (IRYCIS) University of Alcalá 28805 Madrid Spain uah.es; ^2^ Networking Research Center on Bioengineering, Biomaterials & Nanomedicine (CIBER-BBN) 28029 Madrid Spain; ^3^ The Nanomedicine Group Universidad de Cantabria-IDIVAL Avda Herrera Oria s/n 39011 Santander Spain unican.es; ^4^ Departamento de Anatomía y Biología Celular Universidad de Cantabria Santander Spain unican.es; ^5^ Molecular Biology Department Faculty of Medicine Universidad de Cantabria 39011 Santander Spain unican.es

**Keywords:** carbosilane, melanoma, metallodendrimers, norspermidine, polyamines, ruthenium

## Abstract

Metastatic melanoma remains a major therapeutic challenge due to the limited efficacy of current systemic treatments, which is motivating the development of alternative drug delivery strategies. Herein, we report the synthesis and characterization of carbosilane metallodendrimers functionalized with Ru(II) complexes bearing norspermidine‐derived ligands and evaluate their anticancer activity using malignant melanoma as a model of aggressive cancer. In vitro and in vivo studies demonstrate that these macromolecular systems exhibit enhanced antitumour activity compared with their mononuclear ruthenium counterparts. Notably, the second‐generation metallodendrimer produced a remarkable antitumour response in vivo, reducing tumour mass by more than 65% after single administration. These findings indicate that the biological performance of these Ru(II)‐based systems depends not only on the presence of multiple metal centres but also on the nanoscale dendritic architecture, highlighting the potential of metallodendrimers as macromolecular platforms for anticancer therapy.

## 1. Introduction

As one of the leading causes of mortality worldwide, cancer continues to present significant clinical hurdles, presenting an opportunity for the development of novel chemical strategies [1]. Melanoma, although accounting for about 1% of all cancers and less than 10% of skin cancers, is responsible for 75% of skin cancer deaths. It can metastasize to any organ, and the most common organs affected are the skin, subcutaneous tissue, lymph nodes, lungs, liver, central nervous system and bones. Currently, the primary treatment for early stage melanoma is surgical resection, which typically involves extended removal of the primary lesion and radiation therapy. In cases of distal melanoma metastasis or inoperable melanoma, systemic therapies, including immunotherapy, chemotherapy and targeted treatments, are essential for managing the disease [2]. However, conventional treatments are associated with notable limitations, including delayed cancer detection, suboptimal drug delivery, systemic toxicity, elevated costs and numerous adverse drug‐related side effects. These limitations highlight the urgent need for more effective and targeted therapeutic approaches to enhance patient outcomes.

Among the many therapeutic approaches, metal‐based drugs have shown remarkable potential due to their unique chemical properties, including variable oxidation states, coordination flexibility and redox activity [3, 4]. Since the discovery of cisplatin, a wide range of metal ions, including Au(I), Ag(I), Ru(II), Pd(II) and Cu(II), have been explored as alternative agents for skin cancer therapy [5].

One of the key factors governing the biological application of metal‐based systems is the nature of the ligands in the metal coordination sphere. These ligand‐dependent properties directly influence cellular uptake, biodistribution and interactions with biological targets, underscoring the importance of rational ligand design for optimizing the activity and selectivity of metal‐based systems for biological applications. In this context, the antitumour activity of ruthenium (Ru)‐based compounds and their derivatives has been well documented in the literature, particularly owing to their distinctive chemical properties, including redox activity, ligand exchange kinetics and selective accumulation in tumour tissues [6–8]. Recently, we have published the synthesis and characterization of novel Ru(II) complexes with norspermidine‐derived ligands, which present significant anticancer activity against prostate cancer cell lines, particularly PC3 [9]. The incorporation of norspermidine ligands for the posterior coordination of metal complexes offers several advantages: (i) enhanced hydrophobicity of the final compounds, which facilitates cell membrane penetration and promotes efficient cellular internalization; (ii) the norspermidine ligands can mimic natural polyamines that are readily recognized and taken up by cancer cells, leading to increased intracellular accumulation and improving therapeutic efficacy; and (iii) the use of these ligands with two terminal NH_2_ groups per unit allows for the duplication of the number of metal complexes that can be attached to their surface.

However, the clinical application of metal complexes, including cisplatin, is often limited by issues such as systemic toxicity, poor selectivity and the development of drug resistance [10]. To overcome these limitations, nanotechnology has emerged as a powerful strategy to enhance therapeutic efficacy, reduce systemic toxicity and enable targeted drug delivery in melanoma treatment. Nanostructured delivery systems can offer controlled and sustained release, improved bioavailability and selective accumulation in melanoma tissues, thereby enhancing pharmacokinetic profiles and overcoming critical limitations associated with traditional therapeutic agents [11]. In this context, dendrimers have attracted significant interest due to their tuneable size, shape and surface chemistry, which facilitates efficient encapsulation and targeted transport of therapeutic agents [12–14]. Dendritic metallodrugs can be extremely efficient against cancers both in vitro and in vivo [15]. A series of ruthenium arene complexes bearing *para*‐cymene (p‐cymene) or cyclopentadienyl (cp) ligands, attached to the surface of different generations of PPI dendrimers [16, 17] or arylester dendrimers [18], have been shown to be efficient anticancer agents, with half‐maximal inhibitory concentration (IC_50_) values in the micromolar range across various cell lines. Among dendritic architectures, carbosilane dendrimers are particularly notable for biomedical applications, owing to their robust silicon–carbon backbone, structural versatility and high potential for multivalent functionalization [19, 20]. In cancer treatment, a promising synthetic strategy involves the peripheral modification of carbosilane dendritic scaffolds with Ru(II) complexes bearing arene or cyclopentadienyl ligands, coordinated through iminopyridine groups anchored at the dendrimer surface [21–23]. This functionalization approach combines the advantages of dendritic delivery systems, while the carbosilane backbone provides greater flexibility and allows fine‐tuning of the hydrophobic/hydrophilic balance once the multivalent surface is decorated with Ru(II) complexes. This amphiphilic character significantly enhances cellular internalization and preserves the therapeutic potential of metal‐based drugs, which have demonstrated strong anticancer activity in both in vitro and in vivo models across various tumour types.

To validate this approach, we have designed a series of norspermidine‐derived carbosilane metallodendrimers containing *η* ^6^‐p‐cymene ligands coordinated to Ru(II) centres and evaluated their in vivo antimelanoma activity. This novel methodology allows for the preparation of dendritic systems featuring twice the number of metal centres per molecule compared to previously reported examples in the literature [24].

## 2. Materials and Methods

### 2.1. Syntheses

All the reagents used in the different synthetic routes described were purchased from commercial sources and used as received without prior purification (see electrospray ionization [ESI] for experimental details and methodologies of biological assays).

#### 2.1.1. Reagents

*Bis*‐(3‐aminopropylamine) 98%, Sigma‐Aldrich, CAS 56‐18‐8; phthalic anhydride 100.0%, Thermo Scientific, CAS 85‐44‐9; potassium carbonate anhydrous ≥ 99%, Sigma‐Aldrich, CAS 7681‐82‐5; sodium iodide 99% pure anhydrous; Thermo Scientific, CAS 7681‐82‐5; 18‐crown‐6 ≥ 99%, Sigma‐Aldrich, CAS 17455‐13‐9; silica gel 60, 0.04–0.06 mm, Scharlau, CAS 7631‐86‐9; hydrazine hydrate solution 55%, Fluorochem, CAS 7803‐57‐8; 2‐pyridinecarboxaldehyde 99%, Sigma‐Aldrich, CAS 1121‐60‐4; magnesium sulphate anhydrous pure 98%, PanReac‐Applichem, CAS 7487‐88‐9; dichloro(p‐cymene)ruthenium (II) dimer 97%, Fluorochem, CAS 52462‐29‐0. Carbosilane dendrimers bearing a bromine atom on their surface G_n_‐[Br]_m_ (n = 1, m = 4 (**II**); n = 2, m = 8 (**III**)) and N‐bis‐(phthalimidepropyl)amine were prepared by the previously described protocol [25, 26].

#### 2.1.2. Solvents

Acetone ≥ 99.8%, Honeywell, CAS 67‐64‐; n‐hexane 96%, Scharlau, CAS 110‐54‐3; ethyl acetate, Scharlau, CAS 141‐78‐6; methanol ≥ 99.9%, Sigma‐Aldrich, CAS 67‐56‐1; dichloromethane, stabilized with approx. 50 ppm of amylene, Scharlau, CAS 75‐09‐2; chloroform, stabilized with ethanol, Scharlau, CAS 67‐66‐3; toluene, Scharlau, CAS 108‐88‐3; CDCl_3_ 99.8%D, Eurisotop, CAS 865‐49‐6; CD_3_OD 99.8%D, Eurisotop, CAS 811‐98‐3, were prepared. Dry toluene was obtained using a MBraun MB SPS purification system.

#### 2.1.3. Characterization Techniques

*NMR experiments* single‐ and two‐dimensional NMR experiments, including ^1^H NMR, ^13^C{^1^H} NMR, ^1^H–^1^H COSY and ^1^H–^13^C HSQC were performed using a Bruker AVANCE NEO spectrometer operating at 400 MHz equipped with automated locking and shimming systems. ^1^H NMR spectra were collected using 16 scans with a relaxation delay (rd) of 1.0 s and a spectral width (sw) of 8196.7 Hz. For ^13^C{^1^H} NMR experiments, 1024 scans were recorded with a relaxation delay of 2.0 s and a spectral width of 23,809.5 Hz. Two‐dimensional ^1^H–^1^H COSY spectra were obtained using a single scan, a relaxation delay of 2.0 s and a spectral width of 5263.2 Hz. The ^1^H–^13^C HSQC experiments were recorded with 8 scans and a relaxation delay of 1.0 s, using spectral widths of 4854.4 and 24,149.5 Hz, respectively.

Samples were prepared in deuterated solvents (CDCl_3_ and CD_3_OD). Chemical shifts in ^1^H NMR spectra were referenced to the residual solvent signals at *δ* 7.26 ppm for CDCl_3_ and *δ* 3.31 ppm for CD_3_OD, while ^13^C{^1^H} NMR spectra were referenced to the corresponding solvent resonances at *δ* 77.16 ppm (CDCl_3_) and *δ* 49.00 ppm (CD_3_OD).

*Mass spectroscopy* experiments were carried out using a high‐resolution Triple TOF 5600 plus SCIEX spectrometer. The ionization source used was ESI operating in the positive mode, with a capillary voltage of 550 V and a source temperature of 400°C. Mass spectra were acquired using a time‐of‐flight scan (TOF‐MS) over an m/z range of 100–4000 Da. The analysed samples were initially dissolved in dichloromethane (500 µL) and subsequently diluted to 1:75 in methanol. Samples were introduced into the instrument through an Agilent 1100 liquid chromatograph by injecting 2 µL of the sample solution using a flow‐injection analysis (FIA) method. The mobile phase consisted of H_2_O/acetonitrile (25:75, v/v) containing 0.1% formic acid, delivered at a flow rate of 300 µL/min.

*Inductively Coupled Plasma-Optical Emission Spectroscopy (ICP-OES*) was used to determine the percentage of metal centres present in the synthesized complexes using an ICP‐OES Varian‐720‐ES instrument. The samples of each compound were measured by duplicate with the results expressed as the mean and standard deviation. For Ru determination, the calibration line was prepared by dissolving RuCl_3_ in 5% nitric acid (ppb‐trace) and diluting it 1:10 to obtain a stock solution of 10 mg/L. From this stock solution, additional standard solutions (20, 50 and 100 ppb) were prepared for the calibration line. A 2 ppb yttrium solution was used as an internal standard. A Milestone Ethos D microwave Digester with a HPR 1000/6 M rotor was used to prepare the samples. The digestion process involved adding 4 mL HNO_3_ to the samples and heating them in a microwave at 250 W for 5 min, followed by 600 W for 5 min and finally 300 W for 5 min. Sample analysis involved placing them in an automatic injector (power: 1.3 Kw, plasma flow: 15 L/min, auxiliary flow: 1.5, nebulization flow: 1.0 L/min, reading time: 20 s, stability delay: 15 s, delay sampling: 20 s, pump speed: 12 rpm, washing time: 30 s). The selected wavelengths for ruthenium were 240.272, 245.657 and 267.876. The results were expressed as mg (ruthenium)/g (sample). (LDD: 3 ppb and LDC: 8 ppb).

#### 2.1.4. Synthesis of G_1_‐[N[(CH_2_)_3_Pht]_2_]_4_ (1)

To a solution of N‐*bis*‐(phthalimidepropyl)amine (1032.7 mg, 2.64 mmol) in acetone, K_2_CO_3_ (362.85, 2.64), NaI (82.4 mg, 0.55 mmol), 18‐Crown‐6 ether (1.0 mg, 3.78 µmol) and G_1_Br_4_ dendrimer **II** (535.3 mg, 0.55 mmol) were sequentially added. The mixture was stirred at 90°C overnight. After completion of the reaction, the mixture was filtered with silica gel and the solvent was evaporated. The crude reaction mixture was washed twice with hexane. MeOH was then added, and the resulting suspension was heated to reflux until complete dissolution was achieved. The solution was subsequently allowed to cool to room temperature, during which the product precipitated as an oil. Finally, the supernatant was decanted, and the oily product was washed twice with methanol. Residual solvent was removed under vacuum. Compound **1** was isolated as yellow oil (820.4 mg, 67.4%). C_124_H_160_N_12_O_16_Si_5_ (2215.1 g/mol).^**1**^ **H-NMR (CDCl**_**3**_ **):** *δ* (ppm): −0.08 (s, 24H, (**H**_**3**_C)_2_Si‐); 0.44 (m, 8H, ‐SiC**H**_**2**_CH_2_CH_2_CH_2_N‐); 0.53 (m, overlapping of signals, 16H, SiC**H**_**2**_CH_2_CH_2_Si(CH_3_)_2_ and SiCH_2_CH_2_C**H**_**2**_Si(CH_3_)_2_); 1.27 (m, overlapping of signals, 16H, SiCH_2_C**H**_**2**_CH_2_Si(CH_3_)_2_ and ‐SiCH_2_C**H**_**2**_CH_2_CH_2_N‐); 1.39 (m, 8H, ‐SiCH_2_CH_2_C**H**_**2**_CH_2_N‐); 1.81 (m, 16H, ‐NCH_2_C**H**_**2**_CH_2_NPht): 2.38(t, ^3^J_H-H_ = 7.04 Hz, 8H, ‐SiCH_2_CH_2_CH_2_C**H**_**2**_N‐); 2.48 (t, ^3^J_H-H_ = 6.44 Hz, 16H, ‐NC**H**_**2**_CH_2_CH_2_NPht); 3.72 (t, ^3^J_H-H_ = 7.34 Hz, 16H, ‐NCH_2_CH_2_C**H**_**2**_NPht); 7.69 (m, 16H, ‐N(C=O)CCHC**H**‐^Ar^); 7.81 (m, 16H, ‐N(C=O)CC**H**CH‐^Ar^). ^**13**^ **C{** ^**1**^ **H} NMR (CDCl**_**3**_ **):** *δ* −3.10 ((H_3_ **C**)_2_Si‐); 15.79 (‐Si**C**H_2_CH_2_CH_2_CH_2_N‐); 17.89 (Si**C**H_2_CH_2_CH_2_Si(CH_3_)_2_); 18.83 (SiCH_2_ **C**H_2_CH_2_Si(CH_3_)_2_); 20.42 (SiCH_2_CH_2_ **C**H_2_Si(CH_3_)_2_); 22.01 (‐SiCH_2_ **C**H_2_CH_2_CH_2_N‐); 26.22 (‐NCH_2_ **C**H_2_CH_2_NPht); 31.09 (‐SiCH_2_CH_2_ **C**H_2_CH_2_N‐); 36.81 (‐NCH_2_CH_2_ **C**H_2_NPht); 51.77 (‐N**C**H_2_CH_2_CH_2_NPht); 53.65 (‐SiCH_2_CH_2_CH_2_ **C**H_2_N‐); 123.55 (‐N(C=O)C**C**HCH‐^Ar^); 132.45 (‐N(C=O)**C**CHCH‐^Ar^); 134.17 (‐N(C=O)CCH**C**H‐^Ar^); 168.40 (**C**=O). **ESI-TOF (m/z):** [M+2H^+^], Calc.: 1107.55336 uma. Exp.: 1107.55575 uma.

#### 2.1.5. Synthesis of G_2_‐[N[(CH_2_)_3_Pht]_2_]_8_ (2)

The second generation dendrimer **2** was synthesized through the same protocol as that for compound **1**, using the following reagents: N‐*bis*‐(phthalimidepropyl)amine (586.8 mg, 1.5 mmol) in acetone, K_2_CO_3_ (207.3, 1.5), NaI (24.0 mg, 0.16 mmol), 18‐Crown‐6 ether (1.0 mg, 3.78 µmol) and G_2_Br_8_ dendrimer **III** (379.4 mg, 0.16 mmol). Compound **2** was isolated as yellow oil (501.1 mg, 66.1%). C_264_H_356_N_24_O_32_Si_13_ (4743.0 g/mol). ^**1**^ **H-NMR (CDCl**_**3**_ **):** *δ* (ppm): −0.17 (s, 48H, (**H**_**3**_C)_2_Si‐); −0,05 (s, 12H, (**H**_**3**_C)Si‐); 0.44 (m, 16H, ‐SiC**H**_**2**_CH_2_CH_2_CH_2_N‐); 0.52 (m, overlapping of signals, 48H, SiC**H**_**2**_CH_2_CH_2_Si(CH_3_), SiCH_2_CH_2_C**H**_**2**_Si(CH_3_), (CH_3_)SiC**H**_**2**_CH_2_CH_2_Si(CH_3_)_2_) and (CH_3_)SiCH_2_CH_2_C**H**_**2**_Si(CH_3_)_2_); 1.27 (m, overlapping of signals, 40H, SiCH_2_C**H**_**2**_CH_2_Si(CH_3_), (CH_3_)SiCH_2_C**H**_**2**_CH_2_Si(CH_3_)_2_) and ‐SiCH_2_C**H**_**2**_CH_2_CH_2_N‐); 1.38 (m, 16H, ‐SiCH_2_CH_2_C**H**_**2**_CH_2_N‐); 1.78 (m, 32H, ‐NCH_2_C**H**_**2**_CH_2_NPht); 2.37 (m, 16H, ‐SiCH_2_CH_2_CH_2_C**H**_**2**_N‐); 2.47 (m, 32H, ‐NC**H**_**2**_CH_2_CH_2_NPht); 3.69 (m, 32H, ‐NCH_2_CH_2_C**H**_**2**_NPht); 7.68 (m, 32H, ‐N(C=O)CCHC**H**‐^Ar^); 7.79 (m, 32H, ‐N(C=O)CC**H**CH‐^Ar^). ^**13**^ **C{** ^**1**^ **H} NMR (CDCl**_**3**_ **):** *δ* −3.10 ((H_3_ **C**)_2_Si‐); 1.08 ((H_3_ **C**) Si‐); 15.64 (‐Si**C**H_2_CH_2_CH_2_CH_2_N‐); 18.53 (SiCH_2_ **C**H_2_CH_2_Si(CH_3_) and (CH_3_)SiCH_2_ **C**H_2_CH_2_Si(CH_3_)_2_); 20.17 (Si**C**H_2_CH_2_CH_2_Si(CH_3_), SiCH_2_CH_2_ **C**H_2_Si(CH_3_), (CH_3_)Si**C**H_2_CH_2_CH_2_Si(CH_3_)_2_ and (CH_3_)SiCH_2_CH_2_ **C**H_2_Si(CH_3_)_2_); 21.83 (‐SiCH_2_ **C**H_2_CH_2_CH_2_N‐); 26.45 (‐NCH_2_ **C**H_2_CH_2_NPht); 30.98 (‐SiCH_2_CH_2_ **C**H_2_CH_2_N‐); 37.07 (‐NCH_2_CH_2_ **C**H_2_NPht); 51.26 (‐N**C**H_2_CH_2_CH_2_NPht); 53.73 (‐SiCH_2_CH_2_CH_2_ **C**H_2_N‐); 123.15 (‐N(C=O)C**C**HCH‐^Ar^); 132.24 (‐N(C=O)**C**CHCH‐^Ar^); 133.43 (‐N(C=O)CCH**C**H‐^Ar^); 168.60 (**C**=O). **ESI-TOF (m/z):** [M+4H^+^], Calc.: 1185.60648 uma. Exp.: 1185.61497 uma.

#### 2.1.6. Synthesis of G_1_‐[N[(CH_2_)_3_NH_2_]_2_]_4_ (3)

Argon was bubbled through a solution of **1** (638.3 mg, 0.29 mmol) in methanol, before hydrazine monohydrate (348.4 mg, 6.96 mmol) was added. The mixture was stirred at 90°C for 16 h. After completion of the reaction, the solvent was evaporated under vacuum and a gelatinous white solid was obtained. The product was extracted 4 times with dichloromethane at 0°C, and the combined extracts were concentrated in vacuo. The resulting dendrimers were isolated as concentrated residues, since complete drying resulted in irreversible aggregation of the polyamine‐functionalized dendrimer. Compound **3** was obtained as a yellow oil and was stored as a chloroform solution at 5°C (261.9 mg, 76.9%). C_60_H_144_N_12_Si_5_ (1174.3 g/mol). ^**1**^ **H-NMR (CDCl**_**3**_ **):** *δ* (ppm): −0.07 (s, 24H, (**H**_**3**_C)_2_Si‐); 0.47 (m, 8H, ‐SiC**H**_**2**_CH_2_CH_2_CH_2_N‐); 0.54 (m, overlapping of signals, 16H, SiC**H**_**2**_CH_2_CH_2_Si(CH_3_)_2_ and SiCH_2_CH_2_C**H**_**2**_Si(CH_3_)_2_); 1.27 (m, overlapping of signals, 16H, SiCH_2_C**H**_**2**_CH_2_Si(CH_3_)_2_ and ‐SiCH_2_C**H**_**2**_CH_2_CH_2_N‐); 1.43 (m, 8H, ‐SiCH_2_CH_2_C**H**_**2**_CH_2_N‐); 1.59 (m, 16H, ‐NCH_2_C**H**_**2**_CH_2_NH_2_); 1.91 (s, 16H, ‐N**H**_**2**_); 2.38 (t, ^3^J_H-H_ = 7.60 Hz, 8H, ‐SiCH_2_CH_2_CH_2_C**H**_**2**_N‐); 2.45 (t, ^3^J_H-H_ = 7.12 Hz, 16H, ‐NC**H**_**2**_CH_2_CH_2_NH_2_); 2.73 (t, ^3^J_H-H_ = 6.76 Hz, 16H, ‐NCH_2_CH_2_C**H**_**2**_NH_2_). ^**13**^ **C{** ^**1**^ **H} NMR (CDCl**_**3**_ **):** *δ* −3.20 ((H_3_ **C**)_2_Si‐); 15.69 (‐Si**C**H_2_CH_2_CH_2_CH_2_N‐); 17.91 (Si**C**H_2_CH_2_CH_2_Si(CH_3_)_2_); 18.69 (SiCH_2_ **C**H_2_CH_2_Si(CH_3_)_2_); 20.33 (SiCH_2_CH_2_ **C**H_2_Si(CH_3_)_2_); 22.13 (‐SiCH_2_ **C**H_2_CH_2_CH_2_N‐); 30.44 (‐NCH_2_ **C**H_2_CH_2_NH_2_); 30.99 (‐SiCH_2_CH_2_ **C**H_2_CH_2_N‐); 40.81 (‐NCH_2_CH_2_ **C**H_2_NH_2_); 52.24 (‐N**C**H_2_CH_2_CH_2_NH_2_); 53.78 (‐SiCH_2_CH_2_CH_2_ **C**H_2_N‐). **ESI-TOF (m/z):** [M+3H^+^], Calc.: 392.02338 uma. Exp.: 392.02391 uma.

#### 2.1.7. Synthesis of G_2_‐[N[(CH_2_)_3_NH_2_]_2_]_8_ (4)

The second generation dendrimer **4** was synthesized through the same protocol as compound **3**, using the following reagents: **2** (303.3 mg, 0.06 mmol) and hydrazine monohydrate (144.2 mg, 2.88 mmol). Compound **4** was obtained as a yellow oil and was stored as a chloroform solution at 5°C (127.4 mg, 79.8%). C_136_H_324_N_24_Si_13_ (2661.4 g/mol). ^**1**^ **H-NMR (CDCl**_**3**_ **):** *δ* (ppm): −0.10 (s, 48H, (**H**_**3**_C)_2_Si‐); 0,02 (s, 12H, (**H**_**3**_C)Si‐); 0.43 (m, 16H, ‐SiC**H**_**2**_CH_2_CH_2_CH_2_N‐); 0.49 (m, overlapping of signals, 48H, SiC**H**_**2**_CH_2_CH_2_Si(CH_3_), SiCH_2_CH_2_C**H**_**2**_Si(CH_3_), (CH_3_)SiC**H**_**2**_CH_2_CH_2_Si(CH_3_)_2_) and (CH_3_)SiCH_2_CH_2_C**H**_**2**_Si(CH_3_)_2_); 1.24 (m, overlapping of signals, 40H, SiCH_2_C**H**_**2**_CH_2_Si(CH_3_), (CH_3_)SiCH_2_C**H**_**2**_CH_2_Si(CH_3_)_2_) and ‐SiCH_2_C**H**_**2**_CH_2_CH_2_N‐); 1.39 (m, 16H, ‐SiCH_2_CH_2_C**H**_**2**_CH_2_N‐); 1.53 (m, 32H, ‐NCH_2_C**H**_**2**_CH_2_NH_2_); 2.33 (t, ^3^J_H-H_ = 7.58 Hz, 16H, ‐SiCH_2_CH_2_CH_2_C**H**_**2**_N‐); 2.39 (t, ^3^J_H-H_ = 7.18 Hz, 32H, ‐NC**H**_**2**_CH_2_CH_2_NH_2_); 2.67 (t, ^3^J_H-H_ = 6.86 Hz, 32H, ‐NCH_2_CH_2_C**H**_**2**_NH_2_). ^**13**^ **C{** ^**1**^ **H} NMR (CDCl**_**3**_ **):** *δ* −3.19 ((H_3_ **C**)_2_Si‐); 0.97 ((H_3_ **C**)Si‐); 15.10 (‐Si**C**H_2_CH_2_CH_2_CH_2_N‐); 18.70 (Si**C**H_2_CH_2_CH_2_Si(CH_3_), SiCH_2_ **C**H_2_CH_2_Si(CH_3_), SiCH_2_CH_2_ **C**H_2_Si(CH_3_), (CH_3_)Si**C**H_2_CH_2_CH_2_Si(CH_3_)_2_ and (CH_3_)SiCH_2_ **C**H_2_CH_2_Si(CH_3_)_2_); 20.09 ((CH_3_)SiCH_2_CH_2_ **C**H_2_Si(CH_3_)_2_); 22.02 (‐SiCH_2_ **C**H_2_CH_2_CH_2_N‐); 30.98 (‐SiCH_2_CH_2_ **C**H_2_CH_2_N‐)and ‐NCH_2_ **C**H_2_CH_2_NH_2_); 40.94 (‐NCH_2_CH_2_ **C**H_2_NH_2_); 52.16 (‐N**C**H_2_CH_2_CH_2_NH_2_); 53.68 (‐SiCH_2_CH_2_CH_2_ **C**H_2_N‐).

#### 2.1.8. Synthesis of G_1_‐[N[(CH_2_)_3_NCHPh(o‐N)]_2_]_4_ (5)

Under an inert atmosphere, dendrimer **3** (113.0 mg, 0.10 mmol) was dissolved in chloroform in the presence of anhydrous MgSO_4_. The required amount of 2‐pyridinecarboxaldehyde (85.7 mg, 0.80 mmol) was added in three successive portions. Following each addition, an aliquot of the reaction mixture was analysed by ^1^H NMR spectroscopy to determine the degree of peripheral functionalization before the next portion of aldehyde was introduced. The reaction mixture was stirred at room temperature for 15 min. After completion of the reaction, MgSO_4_ was removed by filtration and the solvent was evaporated. Compound **5** was isolated as a brown oil and was stored as a chloroform solution at 5°C (141.5%, 75%). C_108_H_168_N_20_Si_5_ (1887.1 g/mol).^**1**^ **H-NMR (CDCl**_**3**_ **):** *δ* (ppm): −0.08 (s, 24H, (**H**_**3**_C)_2_Si‐); 0.44 (m, 8H, ‐SiC**H**_**2**_CH_2_CH_2_CH_2_N‐); 0.52 (m, overlapping of signals, 16H, SiC**H**_**2**_CH_2_CH_2_Si(CH_3_)_2_ and SiCH_2_CH_2_C**H**_**2**_Si(CH_3_)_2_); 1.26 (m, overlapping of signals, 16H, SiCH_2_C**H**_**2**_CH_2_Si(CH_3_)_2_ and ‐SiCH_2_C**H**_**2**_CH_2_CH_2_N‐); 1.44 (m, 8H, ‐SiCH_2_CH_2_C**H**_**2**_CH_2_N‐); 1.86 (m, 16H, ‐NCH_2_C**H**_**2**_CH_2_N=CH‐); 2.42 (t, ^3^J_H-H_ = 7.74 Hz, 8H, ‐SiCH_2_CH_2_CH_2_C**H**_**2**_N‐); 2.53 (t, ^3^J_H-H_ = 7.00 Hz, 16H, ‐NC**H**_**2**_CH_2_CH_2_N=CH‐); 3.70 (t, ^3^J_H-H_ = 6.93 Hz, 16H, ‐NCH_2_CH_2_C**H**_**2**_N=CH‐); 7.27 (m, 8H, ‐NCHC**H**CHCHC‐^Ar^); 7.70 (m, 8H, ‐NCHCHC**H**CHC‐^Ar^); 7.95 (m, 8H, ‐NCHCHCHC**H**C‐^Ar^); 8.37 (s, 8H, ‐**H**C=N‐); 8.62 (m, 8H, ‐NC**H**CHCHCHC‐^Ar^). ^**13**^ **C{** ^**1**^ **H} NMR (CDCl**_**3**_ **):** *δ* −3.18 ((H_3_ **C**)_2_Si‐); 15.81 (‐Si**C**H_2_CH_2_CH_2_CH_2_N‐); 17.65 (Si**C**H_2_CH_2_CH_2_Si(CH_3_)_2_); 18.74 (SiCH_2_ **C**H_2_CH_2_Si(CH_3_)_2_); 20.30 (SiCH_2_CH_2_ **C**H_2_Si(CH_3_)_2_); 22.03 (‐SiCH_2_ **C**H_2_CH_2_CH_2_N‐); 28.20 (‐NCH_2_ **C**H_2_CH_2_N=CH‐); 31.18 (‐SiCH_2_CH_2_ **C**H_2_CH_2_N‐); 51.80 (‐N**C**H_2_CH_2_CH_2_N=CH‐); 53.75 (‐SiCH_2_CH_2_CH_2_ **C**H_2_N‐); 59.33 (‐NCH_2_CH_2_ **C**H_2_N=CH‐); 121.17 (‐NCHCHCH**C**HC‐^Ar^); 124.56 (NCH**C**HCHCHC‐^Ar^); 136.81 (NCHCH**C**HCHC‐^Ar^); 149.52 (N**C**HCHCHCHC‐^Ar^); 155.14 (NCHCHCHCH**C**‐^Ar^); 162.15 (‐H**C**=N‐).

#### 2.1.9. Synthesis of G_2_‐[N[(CH_2_)_3_NCHPh(o‐N)]_2_]_8_ (6)

The second generation dendrimer **6** was synthesized through the same protocol as compound **5**, using the following reagents: **4** (212.3 mg, 0.08 mmol) and 2‐pyridinecarboxaldehyde (137.1 mg, 1.28 mmol). Compound **6** was isolated as a brown oil and was stored as a chloroform solution at 5°C (246.5 mg, 75.5%). C_232_H_372_N_40_Si_13_ (4086.9 g/mol). ^**1**^ **H-NMR (CDCl**_**3**_ **):** *δ* (ppm): −0.08 (s, 60H, (**H**_**3**_C)Si‐ and (**H**_**3**_C)_2_Si‐); 0.46 (m, 16H, ‐SiC**H**_**2**_CH_2_CH_2_CH_2_N‐); 0.53 (m, overlapping of signals, 48H, SiC**H**_**2**_CH_2_CH_2_Si(CH_3_), SiCH_2_CH_2_C**H**_**2**_Si(CH_3_), (CH_3_)SiC**H**_**2**_CH_2_CH_2_Si(CH_3_)_2_) and (CH_3_)SiCH_2_CH_2_C**H**_**2**_Si(CH_3_)_2_); 1.29 (m, overlapping of signals, 40H, SiCH_2_C**H**_**2**_CH_2_Si(CH_3_), (CH_3_)SiCH_2_C**H**_**2**_CH_2_Si(CH_3_)_2_) and ‐SiCH_2_C**H**_**2**_CH_2_CH_2_N‐); 1.45 (m, 16H, ‐SiCH_2_CH_2_C**H**_**2**_CH_2_N‐); 1.86 (m, 32H, ‐NCH_2_C**H**_**2**_CH_2_N=CH‐); 2.41 (m, 16H, ‐SiCH_2_CH_2_CH_2_C**H**_**2**_N‐); 2.54 (t, ^3^J_H-H_ = 6.8 Hz, 32H, ‐NC**H**_**2**_CH_2_CH_2_N=CH‐); 3.68 (t, ^3^J_H-H_ = 6.7 Hz, 32H, ‐NCH_2_CH_2_C**H**_**2**_N=CH‐); 7.28 (m, 16H, ‐NCHC**H**CHCHC‐^Ar^); 7.70 (m, 16H, ‐NCHCHC**H**CHC‐^Ar^); 7.96 (m, 16H, ‐NCHCHCHC**H**C‐^Ar^); 8.37 (s, 16H, ‐**H**C=N‐); 8.61 (m, 16H, ‐NC**H**CHCHCHC‐^Ar^). ^**13**^ **C{** ^**1**^ **H} NMR (CDCl**_**3**_ **):** *δ* −3.52 ((H_3_ **C**)_2_Si‐); 15.43 (‐Si**C**H_2_CH_2_CH_2_CH_2_N‐); 18.36 (SiCH_2_ **C**H_2_CH_2_Si(CH_3_) and (CH_3_)SiCH_2_ **C**H_2_CH_2_Si(CH_3_)_2_); 20.40 (Si**C**H_2_CH_2_CH_2_Si(CH_3_), SiCH_2_ **C**H_2_CH_2_Si(CH_3_), (CH_3_)Si**C**H_2_CH_2_CH_2_Si(CH_3_)_2_ and (CH_3_)SiCH_2_CH_2_ **C**H_2_Si(CH_3_)_2_); 22.43 (‐SiCH_2_ **C**H_2_CH_2_CH_2_N‐); 28.48 (‐NCH_2_ **C**H_2_CH_2_N=CH‐); 31.25 (‐SiCH_2_CH_2_ **C**H_2_CH_2_N‐); 51.78 (‐N**C**H_2_CH_2_CH_2_N=CH‐); 53.74 (‐SiCH_2_CH_2_CH_2_ **C**H_2_N‐); 59.78 (‐NCH_2_CH_2_ **C**H_2_N=CH‐); 120.90 (‐NCHCHCH**C**HC‐^Ar^); 124.57 (NCH**C**HCHCHC‐^Ar^); 136.83 (NCHCH**C**HCHC‐^Ar^); 149.50 (N**C**HCHCHCHC‐^Ar^); 154.84 (NCHCHCHCH**C**‐^Ar^); 162.26 (‐H**C**=N‐).

#### 2.1.10. Synthesis of G_1_{[N[[(CH_2_)_3_NCHPh(o‐N)]Ru(*η* ^6^‐p‐cymene)Cl_2_]_2_]_4_} (7)

Under an inert atmosphere, [RuCl_2_(*η* ^6^‐p‐cymene)]_2_ (79.6 mg, 0.13 mmol) was dissolved in dry toluene and a solution of **5** (36.2 mg, 0.03 mmol) in the same solvent was added. The reaction mixture was stirred at room temperature for 16 h whereby a precipitate formed. The solvent was removed by filtration in the Büchner funnel, and the product was washed with toluene and dried under vacuum. Compound **7** was isolated as a reddish brown solid (108.7 mg, 83.6%). C_188_H_280_Cl_16_N_20_Ru_8_Si_5_ (4336.6 g/mol). ^**1**^ **H-NMR (CD**_**3**_ **OD):** *δ* (ppm): −0.03 (s, 24H, (**H**_**3**_C)_2_Si‐); 0.59 (m, overlapping of signals, 24H, SiC**H**_**2**_CH_2_CH_2_Si(CH_3_)_2_, SiCH_2_CH_2_C**H**_**2**_Si(CH_3_)_2_ and ‐SiC**H**_**2**_CH_2_CH_2_CH_2_N‐); 1.04 (m, 24H, ‐CH(C**H**_**3**_)_2_^cym^); 1.12 (m, 24H, ‐CH(C**H**_**3**_)_2_^cym^); 1.35 (m, overlapping of signals, 16H, SiCH_2_C**H**_**2**_CH_2_Si(CH_3_)_2_ and ‐SiCH_2_C**H**_**2**_CH_2_CH_2_N‐); 1.56 (m, 8H, ‐SiCH_2_CH_2_C**H**_**2**_CH_2_N‐); 1.80 (m, 16H, ‐NCH_2_C**H**_**2**_CH_2_N=CH‐); 2.28 (m, 8H, ‐SiCH_2_CH_2_CH_2_C**H**_**2**_N‐); 2.31 (s, 24H, **H**_**3**_CC‐^cym^); 2.44 (m, 16H, ‐NC**H**_**2**_CH_2_CH_2_N=CH‐); 2.66 (m, 8H, ‐C**H**(CH_3_)_2_^cym^); 4.43 (m, 8H, ‐NCH_2_CH_2_C**H**_**2**_N=CH‐); 4.75 (m, 8H, ‐NCH_2_CH_2_C**H**_**2**_N=CH‐); 5.89 (m, 16H, ‐C**H** ^Ar^CCH_3_^cym^); 6.18 (m, 16H, ‐C**H** ^Ar^CCH(CH_3_)_2_^cym^); 7.79 (m, 8H, ‐NCHCHC**H**CHC‐^Ar^); 8.19 (m, overlapping of signals, 16H, ‐NCHCHCHC**H**C‐^Ar^ and ‐NCHC**H**CHCHC‐^Ar^); 8.82 (s, 8H, ‐**H**C=N‐); 9.48 (m, 8H, ‐NC**H**CHCHCHC‐^Ar^). ^**13**^ **C{** ^**1**^ **H} NMR (CD**_**3**_ **OD):** *δ* −3.67 ((H_3_ **C**)_2_Si‐); 17.11 (‐CH(**C**H_3_)_2_^cym^); 18.97 (overlapping of signals, Si**C**H_2_CH_2_CH_2_Si(CH_3_)_2_), SiCH_2_CH_2_ **C**H_2_Si(CH_3_)_2_) and ‐Si**C**H_2_CH_2_CH_2_CH_2_N‐); 21.73 (overlapping of signals, SiCH_2_ **C**H_2_CH_2_Si(CH_3_)_2_), ‐SiCH_2_ **C**H_2_CH_2_CH_2_N‐, ‐SiCH_2_CH_2_ **C**H_2_CH_2_N‐ and ‐NCH_2_ **C**H_2_CH_2_N=CH‐); 22.46 (‐CH(**C**H_3_)_2_^cym^); 23.11 (‐SiCH_2_CH_2_CH_2_ **C**H_2_N‐ and ‐N**C**H_2_CH_2_CH_2_N=CH‐); 31.50 (‐**C**H(CH_3_)_2_^cym^); 64.98 (‐NCH_2_CH_2_ **C**H_2_N=CH‐); 84.93 (‐**C**H^Ar^CCH_3_^cym^); 86.56 (‐**C**H^Ar^CCH_3_^cym^); 89.07 (‐**C**H^Ar^CCH(CH_3_)_2_^cym^); 106.07 (‐CH^Ar^ **C**CH(CH_3_)_2_^cym^); 107.17 (‐CH^Ar^ **C**CH_3_^cym^); 127.11 (‐NCHCH**C**HCHC‐^Ar^); 130.19 (‐NCH**C**HCHCHC‐^Ar^); 140.92 (‐NCHCHCH**C**HC‐^Ar^); 155.76 (‐N**C**HCHCHCHC‐^Ar^); 157.53 (‐NCHCHCHCH**C**‐^Ar^); 170.61 (‐H**C**=N‐). **ICP-OES (%):** Cal.: Ru, 18.64. Exp.: 15.52 ± 0.56.

#### 2.1.11. Synthesis of G_2_{[N[[(CH_2_)_3_NCHPh(o‐N)]Ru(*η* ^6^‐p‐cymene)Cl_2_]_2_]_8_} (8)

The second generation metallodendrimer **8** was synthesized through the same protocol as complex **7**, using the following reagents: **6** (26.9 mg, 0.01 mmol) and [RuCl_2_(*η* ^6^‐p‐cymene)]_2_ (55.1 mg, 0.09 mmol). Compound **8** was isolated as a reddish‐brown solid (72.6 mg, 89.8%). C_393_H_595_Cl_32_N_40_Ru_16_Si_13_ (8985.0 g/mol). ^**1**^ **H-NMR (CD**_**3**_ **OD):** *δ* (ppm): 0.03 (s, 60H, (**H**_**3**_C)Si‐ and (**H**_**3**_C)_2_Si‐); 0.60 (m, overlapping of signals, 64H, SiC**H**_**2**_CH_2_CH_2_Si(CH_3_), SiCH_2_CH_2_C**H**_**2**_Si(CH_3_), (CH_3_)SiC**H**_**2**_CH_2_CH_2_Si(CH_3_)_2_), (CH_3_)SiCH_2_CH_2_C**H**_**2**_Si(CH_3_)_2_ and ‐SiC**H**_**2**_CH_2_CH_2_CH_2_N‐); 1.00 (m, 48H, ‐CH(C**H**_**3**_)_2_^cym^); 1.14 (m, 48H, ‐CH(C**H**_**3**_)_2_^cym^); 1.37 (m, overlapping of signals, 88H, SiCH_2_C**H**_**2**_CH_2_Si(CH_3_), (CH_3_)SiCH_2_C**H**_**2**_CH_2_Si(CH_3_)_2_, ‐SiCH_2_C**H**_**2**_CH_2_CH_2_N‐, ‐SiCH_2_CH_2_C**H**_**2**_CH_2_N‐ and ‐NCH_2_C**H**_**2**_CH_2_N=CH‐); 1.91 (m, 16H, ‐SiCH_2_CH_2_CH_2_C**H**_**2**_N‐); 2.05 (m, 32H, ‐NC**H**_**2**_CH_2_CH_2_N=CH‐); 2.25 (s, 48H, **H**_**3**_CC‐^cym^); 2.66 (m, 16H, ‐C**H**(CH_3_)_2_^cym^); 4.08 (m, 16H, ‐NCH_2_CH_2_C**H**_**2**_N=CH‐); 4.22 (m, 16H, ‐NCH_2_CH_2_C**H**_**2**_N=CH‐); 5.84 (m, 32H, ‐C**H** ^Ar^CCH_3_^cym^); 6.07 (m, 16H, ‐C**H** ^Ar^CCH(CH_3_)_2_^cym^); 6.17 (m, 16H, ‐C**H** ^Ar^CCH(CH_3_)_2_^cym^); 7.77 (m, 16H, ‐NCHCHC**H**CHC‐^Ar^); 8.16 (m, overlapping of signals, 32H, ‐NCHCHCHC**H**C‐^Ar^ and ‐NCHC**H**CHCHC‐^Ar^); 8.66 (s, 16H, ‐**H**C=N‐); 9.47 (m, 16H, ‐NC**H**CHCHCHC‐^Ar^). ^**13**^ **C{** ^**1**^ **H} NMR (CD**_**3**_ **OD):** *δ* −2.29 ((H_3_ **C**)Si‐ and (H_3_ **C**)_2_Si‐); 13.03 (‐Si**C**H_2_CH_2_CH_2_CH_2_N‐); 17.73 (‐CH(**C**H_3_)_2_^cym^); 19.11 (overlapping of signals, (Si**C**H_2_CH_2_CH_2_Si(CH_3_), SiCH_2_ **C**H_2_CH_2_Si(CH_3_), SiCH_2_CH_2_ **C**H_2_Si(CH_3_), (CH_3_)Si**C**H_2_CH_2_CH_2_Si(CH_3_)_2_, (CH_3_)SiCH_2_ **C**H_2_CH_2_Si(CH_3_)_2_, (CH_3_)SiCH_2_CH_2_ **C**H_2_Si(CH_3_)_2_ and ‐SiCH_2_ **C**H_2_CH_2_CH_2_N‐); 20.60 (‐CH(**C**H_3_)_2_^cym^); 21.64 (‐CH(**C**H_3_)_2_^cym^); 24.49 (‐SiCH_2_CH_2_CH_2_ **C**H_2_N‐ and ‐N**C**H_2_CH_2_CH_2_N=CH‐); 29.48 (‐SiCH_2_CH_2_ **C**H_2_CH_2_N‐ and ‐NCH_2_ **C**H_2_CH_2_N=CH‐); 31.06 (‐**C**H(CH_3_)_2_^cym^); 71.20 (‐NCH_2_CH_2_ **C**H_2_N=CH‐); 84.35 (‐**C**H^Ar^CCH(CH_3_)_2_^cym^); 85.38 (‐**C**H^Ar^CCH_3_^cym^); 87.37 (‐**C**H^Ar^CCH(CH_3_)_2_^cym^); 102.49 (‐CH^Ar^ **C**CH(CH_3_)_2_^cym^); 106.91 (‐CH^Ar^ **C**CH_3_^cym^); 128.44 (‐NCHCH**C**HCHC‐^Ar^ and ‐NCH**C**HCHCHC‐^Ar^); 139.61 (‐NCHCHCH**C**HC‐^Ar^); 155.71 (‐N**C**HCHCHCHC‐^Ar^); 156.06 (‐NCHCHCHCH**C**‐^Ar^); 167.24 (‐H**C**=N‐). **ICP-OES (%):** Cal.: Ru, 17.84. Exp.: 14.00 ± 0.84.

### 2.2. Biological Assays

#### 2.2.1. Cell Culture

Murine melanoma cells (B16‐F10 from Innoprot Company RRID: CVCL_0159) were cultured in Iscove’s modified Dulbecco’s medium (IMDM) supplemented with 10% foetal bovine serum (FBS) and 100 μg/mL gentamicin and maintained at 37°C in a humidified incubator with 5% CO_2_.

#### 2.2.2. In Vitro Studies

Cell viability of the three dendrimers was assessed using the Trypan Blue exclusion assay, which distinguishes live cells from dead ones based on membrane integrity. Cells were incubated with different concentrations of the compounds (ranging from 2 to 40 µM) for 24 h. After treatment, cells were collected by centrifugation and resuspended in phosphate‐buffered saline (PBS). The pellet was mixed with a 0.4% Trypan Blue solution (Thermo Fisher Scientific, USA) at a 1:1 ratio. After a short incubation, the samples were loaded onto a Neubauer chamber and analysed under a light microscope. The proportion of viable cells was calculated as a percentage of the total number of cells. In addition, cell morphology and phenotype were examined through phase‐contrast imaging using a Nikon Eclipse Ti‐U Inverted Fluorescence Microscope.

Confocal microscopy images: Melanoma cells were incubated for 24 h with each compound at its IC_50_ concentration. Cells were then stained with DAPI to label viable nuclei and with propidium iodide (PI) to label nonviable cells and imaged using a Nikon A1R confocal microscope.

#### 2.2.3. Protocol to Determine the [Ru] in Cells Treated

Three types of samples were processed for ICP‐OES analysis: (i) culture medium, (ii) supernatant recovered after cell incubation and (iii) cell pellets. *Digestion of Medium and Supernatant Samples:* For the medium and supernatant, 0.5 mL of liquid sample was mixed with 6 mL of ppb‐trace–grade nitric acid. The samples were subjected to microwave digestion at 200°C for 20 min. After digestion, the resulting solutions were diluted to a final volume of 50 mL with Milli‐Q water. *Preparation and Digestion of Cellular Samples*: Falcon tubes containing the cellular residues were first weighed, frozen, and subsequently lyophilized to completely remove residual water. After lyophilization, the tubes were weighed again to determine the amount of water removed and thus estimate the volume of supernatant retained within the cellular material. The resulting solid fraction was recovered by three successive washes with 2 mL of ppb‐trace nitric acid each (total of 6 mL per sample) and digested under the same microwave conditions as the liquid samples. The digested samples were then diluted to 25 mL with Milli‐Q water. *Ruthenium Quantification*: The ruthenium concentration in the medium and supernatant was calculated using the expression: μgRu/mL = C × 0.1 where C is the concentration determined by ICP‐OES in μg/L. The total amount of ruthenium recovered from the cellular residues and remaining supernatant was calculated as μg Ru_total = C × 0.025, where C is the concentration determined by ICP‐OES in μg/L. The amount of ruthenium corresponding to residual supernatant adhering to the cell samples was determined using: μg Ru_supernatant =  *C*_*M*_ × (*P*_*i*_ − *P*_*f*_), where  *C*_*M*_ is the ruthenium concentration in the supernatant for each experimental condition, *P*_*i*_ is the initial weight of the tube before lyophilization, and *P*_*f*_ is the weight after lyophilization. Finally, the ruthenium content corresponding exclusively to the cells was calculated by subtracting the ruthenium attributable to residual supernatant from the total recovered in the digestion of the solids: μg Ru_cells = μg Ru_total − μg Ru_supernatant.

#### 2.2.4. Preclinical Experiments

In vivo experiments were carefully designed and conducted to minimize the use of animals and ensure their welfare. Ethical approval for all animal procedures was obtained prior to the research from the Gobierno de Cantabria, Consejería de Medio Rural, Pesca y Alimentación (Accreditation No.: PI‐05‐23). The animals were housed under a 12‐h light/dark cycle with ad libitum access to food and water. All procedures involving animals were carried out in compliance with the European directive 2010/63/EU and institutional guidelines for the care and use of laboratory animals. Local intratumoral assays were conducted by subcutaneously injecting 1 × 10^5^ B16‐F10 cells, suspended in 10 μL of PBS supplemented with antibiotics, into CD1 neonatal mice, following the protocol described previously [27]. Seven days after cell implantation, the animals were randomly assigned to three treatment groups: PBS (control), **7** and **8**, each administered at a dose of 10 mg/kg. Mice were sacrificed 48 h after treatment, and tumour masses were excised and weighed.

## 3. Results and Discussion

### 3.1. Synthesis and Structural Characterization of the Novel Ruthenium Carbosilane Metallodendrimers

Based on the synthetic strategy previously developed in our research group for mono‐ and bimetallic systems, first‐ and second‐generation metallodendrimers were synthesized following the protocol reported for the preparation of the so‐called dendritic model Et_3_Si(CH_2_)_3_[N[[(CH_2_)_3_NCHPh(o‐N)]Ru(*η* ^6^‐p‐cymene)Cl_2_]_2_] (**I**) (see Scheme 1) [9]. Accordingly, the precursor dendrimers selected for the preparation of dendritic norspermidine ligands, a structural analogue of the polyamine spermidine (SPD), were carbosilane dendrimers bearing bromine functionalities at the periphery, G_n_‐[Br]_m_ (n = 1, m = 4 (**II**); n = 2, m = 8 (**III**)) [25].

**Scheme 1:**
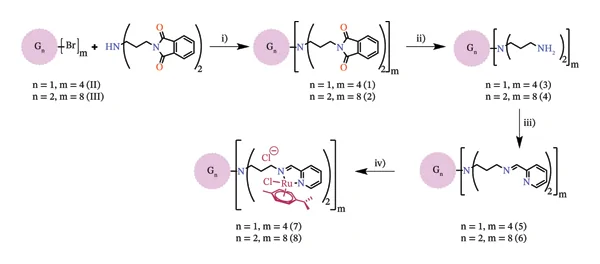
The synthesis procedure of the ruthenium (II) metallodendrimers (i) NaI, K_2_CO_3_, 18‐Crown‐6, acetone, 90°C, 16 h. (ii) Hydrazine, MeOH, 90°C, 16 h; (iii) 2‐pyridinecarboxaldehyde, MgSO_4_, DCM, rt. 15 min (iv) [RuCl_2_(*η* ^6^‐p‐cymene)]_2_, toluene, rt, 16 h.

N‐alkylation of G_n_‐[Br]_m_ dendrimers with an excess of a norspermidine derivative, featuring phthalimide‐protected primary amino groups (**I**) previously described in the bibliography [26], was carried out under basic conditions to obtain the dendritic derivates G_1_{[N[(CH_2_)_3_Pht]_2_]_4_} (**1**) and G_2_{[N[(CH_2_)_3_Pht]_2_]_8_} (**2**). Alkylation was confirmed by ^1^H‐NMR spectroscopy through the disappearance of the C**H**_2_Br signal of the precursor dendrimers at 3.42 ppm and the appearance of resonances corresponding to the newly formed –C**H**_2_N(CH_2_)_2_ moiety at 2.38 ppm, along with aromatic signals from the phthalimide ring at 7.64 and 7.74 ppm (spectroscopic data in Figures S1–S4). Minor additional resonances observed in the SiMe region (around 0 ppm), together with signals detected by ESI‐MS, suggest the presence of a small fraction of structurally defective species (2%–5%). These defects most likely originate from the hydrosilylation and alkylation steps involved in the synthesis of the carbosilane dendritic scaffolds, as previously reported for related carbosilane dendrimers [28, 29]. The presence of these defects is slightly more evident for the second‐generation dendrimer, which can reasonably be attributed to the higher steric congestion around the peripheral reactive sites as the dendrimer generation increases, making complete functionalization progressively more challenging. The ESI‐MS spectra exhibit several minor ions whose m/z values could be consistent with species containing terminal –CH_2_–I groups (see S17‐S20). However, no corresponding ^1^H NMR resonance attributable to the –CH_2_–CH_2_–I fragment was observed, indicating that the available NMR data do not support the presence of such species in the isolated compounds. Therefore, an alternative explanation is proposed in which these ions are formed during the ESI‐MS ionization process, possibly through a Hofmann elimination followed by iodide addition from residual iodide originating from the excess NaI employed in the N‐alkylation reaction.

Subsequent deprotection of the phthalimide groups in MeOH as a solvent using an excess of hydrazine provided the primary amines affording G_1_{[N[(CH_2_)_3_NH_2_]_2_]_4_ (**3**) and G_2_[N[(CH_2_)_3_NH_2_]_2_]_8_ (**4**). Complete solvent removal was avoided, as the high density of peripheral primary amino groups promotes intermolecular aggregation, leading to materials that are difficult to redisperse. The deprotection was confirmed by ^1^H‐NMR, with the disappearance of the aromatic signals of the phthalimide groups and the shift of the signal ‐C**H**_**2**_NPht from 3.72 ppm to a signal for ‐C**H**_**2**_NH_2_ at 2.72 ppm (Figure S5–S8). The condensation reaction of dendrimers **3** and **4** with 2‐pyridinecarboxaldehyde in the presence of anhydrous MgSO_4_ was performed by adding the aldehyde in successive portions. The progress of the reaction was monitored after each addition by ^1^H NMR spectroscopy, and the reaction was considered complete when the Kaiser test was negative and the ^1^H NMR spectrum showed an excess of approximately 1% of unreacted aldehyde, ensuring complete consumption of the primary amine groups. The ^1^H NMR spectra showed the appearance of the imine signal ‐**H**C=N‐ at 8.37 ppm and the methylene C**H**_**2**_N=CH‐ signal at 3.70 ppm (Figure S9–S12). After purification, the first‐ and second‐generation dendrimers G_1_‐[N[(CH_2_)_3_NCHPh(*o*‐N)]_2_]_4_ (**5**) and G_2_‐[N[(CH_2_)_3_NCHPh(*o*‐N)]_2_]_8_ (**6**), bearing surface Schiff base moieties, were obtained as oils and stored as chloroform solutions at 5°C. These systems were designed to act as ligands for coordination with Ru(II) complexes.

Finally, the reaction of dendrimers **5** and **6** with the ruthenium precursor [Ru(*η* ^6^‐p‐cymene)Cl_2_]_2_ was carried out in a ratio of branch:[Ru] of 1:1.1 led to metallodendrimers G_1_{[N[[(CH_2_)_3_NCHPh(*o*‐N)]Ru(*η* ^6^‐p‐cymene)Cl_2_]_2_]_4_} (**7**) and G_2_{[N[[(CH_2_)_3_NCHPh(*o*‐N)]Ru(*η* ^6^‐p‐cymene)Cl_2_]_2_]_8_} (**8**) as a red solids with 8 and 16 metallic centres per molecule (Figure 1). Coordination of the metal centre was confirmed by analysing the resonance pattern of the arene ring and protons of the methylene close to the imine fragment (two split multiplets around 4.24 and 4.66 ppm) in the ^1^H‐NMR spectrum (Figure 2), which is indicative of the presence of a stereogenic centre in the ruthenium (Figures S13–S16). Moreover, ^1^H NMR and 2D‐NMR spectra still exhibited low‐intensity resonances attributable to traces of the free ruthenium dimer after completion of the reaction, whereas no signals corresponding to uncoordinated iminopyridine ligands were detected. The persistence of these traces of the ruthenium precursor is most likely related to the minor population of structurally defective dendritic species present in the precursor dendrimers, which reduces the actual number of accessible coordination sites with respect to the theoretical dendrimer functionality. In agreement with this observation, the experimental Ru contents determined by ICP‐OES in both metallodendrimers **7** and **8** (15.52 ± 0.56 and 14.00 ± 0.54%, respectively) were consistently lower than the theoretical values calculated for defect‐free dendritic structures. Finally, these ruthenium dendrimers exhibit the same coordination mode as dendritic model **I** previously reported, showing high stability in D_2_O after 24 h in solution [9, 24] (see Figure S23).

**FIGURE 1:**
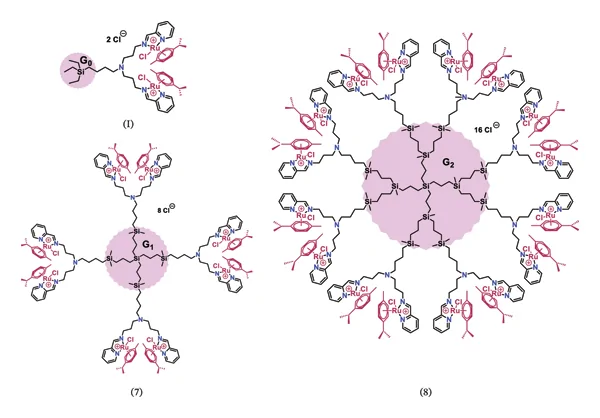
Molecular representation of the proposed structures for dendritic model **I** and the first and second generation metallodendrimers **7** and **8.**

**FIGURE 2:**
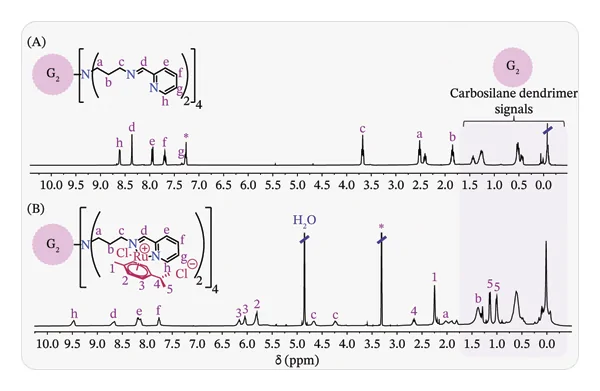
(A) ^1^H‐NMR 400 MHz spectra of G_2_‐[N[(CH_2_)_3_NCHPh(*o*‐N)]_2_]_8_ **(5)** in CDCl_3_ (∗) and (B) G_2_{[N[[(CH_2_)_3_NCHPh(o‐N)]Ru(*η* ^6^‐p‐cymene)Cl_2_]_2_]_8_} **(8)** in CD_3_OD (∗).

### 3.2. Cytotoxic Effects on Malignant Melanoma Cells

Subsequently, the in vitro anticancer activity of the new metallodendrimers **7** and **8**, the dendritic model **I**, and the previously reported monometallic ruthenium complex G_0_‐[[NCPh(*o*‐N)Ru(*η* ^6^‐p‐cymene)Cl]Cl] (**IV)** [24], developed by our research group, was evaluated against the murine malignant melanoma cell line (B16F10) using the Trypan Blue exclusion assay. The monometallic complex (**IV)**, which possesses the same ruthenium coordination sphere as the metallodendrimers, was incorporated into the study as a molecular reference to enable a direct assessment of the contribution of the multimetallic dendritic architecture to the observed biological activity. Melanoma cells were incubated for 24 h with compounds **I**, (**IV)**, **7** and **8** at different concentrations to determine their half‐maximal inhibitory concentration (IC_50_). Viability results were validated by phase‐contrast microscopy, which revealed a marked decrease in the population of viable cells after treatment (see Figure 3B). Morphologically, treated cultures exhibited hallmark cytotoxic phenotypes, including cell rounding and shrinkage, loss of processes, detachment, membrane blebbing and increased cellular debris, together with a rise in PI‐positive nuclei consistent with loss of membrane integrity and cell death (Figure 3C).

**FIGURE 3:**
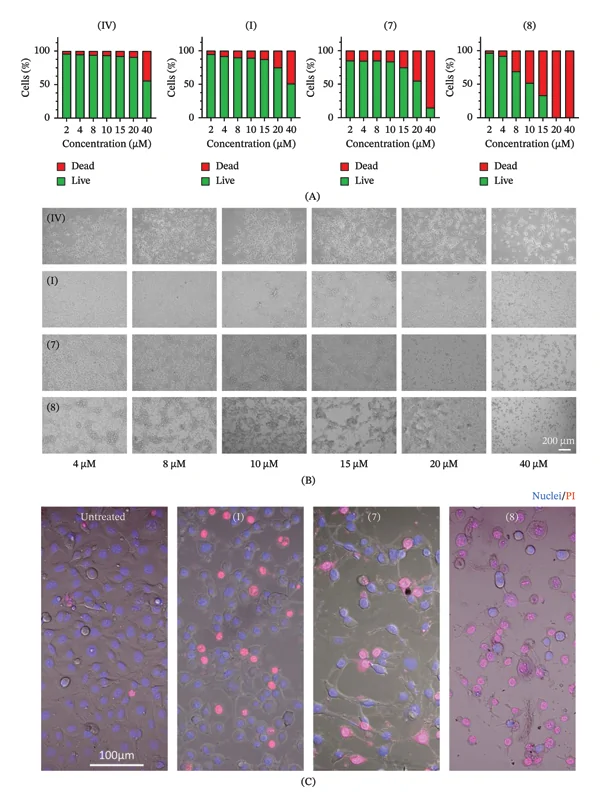
In vitro studies. (A) Dose–response cytotoxicity of compound **I** and metallodendrimers **7** and **8** in B16F10 melanoma cells after 24 h, measured by the Trypan Blue exclusion assay. (B) Phase‐contrast micrographs of melanoma cells exposed to increasing concentrations of **I**, **7** and **8** (scale bar, 200 µm). (C) Confocal fluorescence images of melanoma cells treated with compound **I** and metallodendrimers **7** and **8**; nuclei of viable cells are stained with DAPI (blue) and dead cells with propidium iodide, PI (magenta) (scale bar, 100 µm).

The cytotoxicity of compounds **IV** and **I**, containing one and two Ru(II) centres, respectively, revealed only a modest improvement upon doubling the number of metal centres. The monometallic compound **IV** exhibited an IC_50_ value of 54.6 ± 3.6 μM, whereas the bimetallic compound **I** showed an IC_50_ of 42.5 μM, corresponding to only a 1.3‐fold enhancement in activity. In contrast, the first‐generation metallodendrimer **7** exhibited a lower IC_50_ value of 23.7 ± 2.8 μM, while the second‐generation metallodendrimer **8** further reduced the IC_50_ to 11.2 ± 1.5 μM. When the amount of Ru present at the IC_50_ concentration of each compound was considered, values of 5.5, 8.8, 19.1 and 18.1 mg Ru L^−1^ were obtained for the mono (**IV**)‐, bi (**I**)‐, eight (**7**) and sixteen (**8**) metal centres systems, respectively. Taking the monometallic complex as reference, these values correspond to 1.00‐, 1.50‐, 3.32‐ and 3.25‐fold Ru concentrations, respectively. These results indicate that the decrease in molar IC_50_ observed with an increasing number of metal centres is not accompanied by a proportional enhancement in cytotoxic activity when the Ru concentration present at the corresponding IC_50_ of each system is considered, as an increase in Ru concentration is required to achieve the cytotoxic effect observed for the multimetallic systems. However, this increase does not appear to scale linearly with the number of metal centres, as particularly evidenced by dendritic systems **7** and **8**, which showed comparable Ru concentrations at their respective IC_50_ values (19.1 and 18.1 mg Ru L^−1^), despite their different molar IC_50_ values (23.7 and 11.2 µM, respectively). Overall, these findings suggest that the in vitro cytotoxic response cannot be directly correlated with either the number of Ru centres present at the IC_50_ concentration, indicating that additional structural and physicochemical features of the systems may also contribute to their biological activity. These findings suggest that the Ru content alone may not fully account for the observed differences in biological activity and that other features associated with the dendritic structure should also be considered. In this context, the dendritic architecture may contribute to the biological response through the nanoscale organization and multivalent presentation of the Ru(II) complexes. While these features do not provide a clear advantage in the in vitro assays when Ru content is considered, their contribution may become particularly relevant under in vivo conditions, as demonstrated by the results presented in Table 1.

**TABLE 1:** Antitumoural activity of metal complexes **I**, **IV**, **7** and **8** in the B16F10 cell line after 24 h of treatment, expressed as IC_50_ values per dendritic system (μM and mg compound L^−1^) together with the corresponding ruthenium concentration at the IC_50_ ([Ru], mg Ru L^−1^).

Compound	Ru atoms per compound	IC_50_ (μM)	IC_50_ (mg L^−1^)	[Ru]IC_50_ (mgRu L^−1^)
**IV**	1	54.6 ± 3.6	31.0	5.5
**I**	2	42.5 ± 1.9	46.4	8.8
**7**	8	23.7 ± 2.8	102.8	19.1
**8**	16	11.2 ± 1.5	100.8	18.1

To assess the internalization of the dendritic systems in B16F10 cells as qualitative proof of concept, cellular uptake was monitored by ICP‐OES. For this purpose, cells were treated with a subtoxic concentration of systems **I** (20), **7** (15) and **8** (8 µM) for 24 h. After the exposure period, the cells were centrifuged at 1200 rpm for 5 min. Both the resulting supernatant and the treated cell pellets were subsequently analysed by ICP‐OES. Ruthenium was detected in the cell pellets following treatment with all compounds, confirming their cellular internalization. These findings support the ability of the Ru‐containing carbosilane systems to enter B16F10 cells and are consistent with their observed biological activity.

### 3.3. Antitumoural Activity on Preclinical Models

Our next step was to explore the in vivo effects of the treatments. For this proof‐of‐concept study, we selected a robust and widely validated [27, 30, 31] preclinical model of malignant melanoma, which involves transplanting malignant melanoma tumour cells into immunocompetent mice. The resulting melanoma tumours are pigmented, solid masses, allowing for easy monitoring and isolation, which enhances result reproducibility. Intratumoral delivery strategy has been investigated in patients with advanced melanoma using a variety of agents, including chemotherapeutics, immunomodulators and tumour‐specific vaccines [32]. The treatment involves the direct administration of therapeutic agents into the tumour site.

A dose of 10 mg/kg was selected based on previous in vivo studies using similar metallodendrimers, which demonstrated both tolerability and therapeutic potential in other cancer models [23]. These prior findings supported the use of the same dosage in the present melanoma model to preliminarily assess biocompatibility and short‐term biological effects following local administration. In this study, each treatment group received a single injection of either the metallodendrimer formulation (10 mg/kg) or PBS as a negative control. At this dose, based on the Ru content determined by ICP‐OES, the metallodendrimer formulations contained 18.7%, 17.7%, 15.5% and 14.0% (w/w) ruthenium for compounds **I**, **IV**, **7** and **8**, respectively. Two days after treatment (Day 10 post‐transplant), the mice were sacrificed, and tumours were harvested for mass quantification. A reduction in tumour size compared to PBS‐treated controls was interpreted as indicative of an antitumour effect (Figure 4).

**FIGURE 4:**
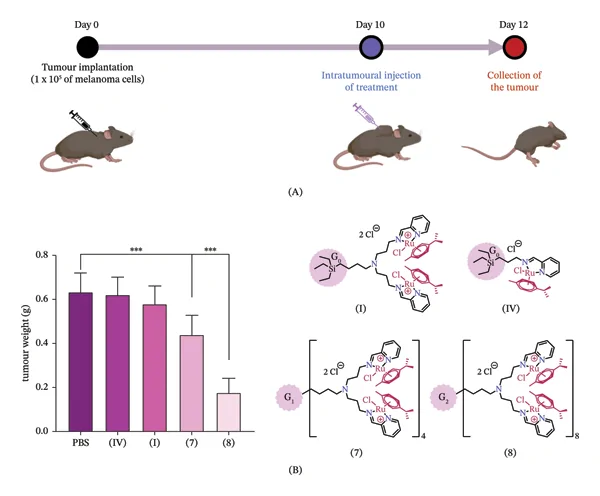
In vivo studies. (A) Schematic representation of the in vivo experimental timeline. (B) Tumour weight at the time of collection. This histogram illustrates the efficacy of different intratumoral treatments, with PBS‐injected mice serving as the control group.

The promising in vitro activity of the metallodendrimers was further corroborated in the in vivo melanoma model (Figure 4). Despite these relatively small differences in the Ru content, compounds **IV** and **I** produced only a limited effect on tumour growth compared with the PBS‐treated control group, whereas treatment with the first‐generation metallodendrimer **7** yielded a substantial 33% reduction in tumour mass. Furthermore, the second‐generation metallodendrimer **8** exhibited the highest antitumour efficacy, reducing tumour mass by more than 65% relative to the control group. The percentage of tumour reduction observed in the present study is consistent with previously reported outcomes for other ruthenium(II)‐based complexes, whereby several recent studies have reported significant antitumour activity of ruthenium(II) complexes in melanoma models [33–35]. Tavares and coworkers evaluated the anti‐melanoma properties of a series of naphthoquinone‐based ruthenium(II) complexes [35]. In their in vivo assays, a marked decrease in tumour size and mass (88.42%) was achieved after a 5‐day treatment regimen. Nevertheless, the animals receiving the compound experienced notable weight loss, which the authors associated with treatment‐induced bone‐marrow aplasia. Meanwhile, Xu and coworkers have demonstrated the therapeutic potential of half‐sandwich ruthenium(II) complexes through the *ex vivo* treatment of B16F10 tumour cells [36]. In this approach, cells were exposed to the ruthenium‐based dendritic system at the IC_50_ concentration for 24 h and subsequently implanted subcutaneously into mice. This strategy resulted in approximately a 60% reduction in tumour growth compared to the control group at 21 days post‐transplantation. In another example, Batista and coworkers achieved similar therapeutic outcomes following daily administration of a monometallic ruthenium complex at 5.0 mg·kg^−1^ body weight over five consecutive days using the B16F10 murine melanoma model [37].

These previous works primarily evaluated mononuclear ruthenium platforms or single‐metal organometallic species [35–37]. However, the carbosilane‐based multimetallic scaffold explored here enables modular tuning of multiple metal centres and ligand environments, potentially enhancing potency, selectivity and pharmacokinetic properties. Remarkably, metallodendrimers **7** and **8** described in this work demonstrated a rapid and pronounced onset of action. Following a single treatment, a conspicuous reduction in tumour mass was observed merely two days postadministration (Day 10 post‐transplant). In comparison with previously reported ruthenium‐based systems, the dendritic ruthenium(II) platforms described herein provoke substantial antitumour responses following a single in vivo administration.

The combined in vitro and in vivo results suggest that the antitumour performance of these Ru(II)‐based systems depends not only on the number of metal centres but also on the size and architecture of the dendritic scaffold. The progressive improvement observed in in vivo results with increasing dendrimer generation highlights the importance of nanoscale structural design in modulating the biological behaviour of these metallodendrimers. Instead, the results suggest that the dendritic architecture also contributes to the enhanced biological response, likely through the nanoscale organization and multivalent presentation of the Ru(II) complexes that could promote tumour accumulation via the enhanced permeability and retention effect, driven by enhanced vascular permeability and impaired lymphatic drainage [38]. In addition, the cytotoxic response may be influenced by the hydrophilic–hydrophobic balance of the system, which governs membrane interactions and cellular uptake, potentially accounting for the higher cytotoxicity observed for the second‐generation dendrimer **(8).** The rapid and substantial reduction in tumour burden achieved within the relatively short treatment period, particularly for the second‐generation metallodendrimer, provides encouraging evidence of the therapeutic potential of these systems. Collectively, these findings support the continued development and further preclinical evaluation of ruthenium‐based dendritic platforms as candidates for melanoma treatment.

## 4. Conclusions

In conclusion, the new ruthenium(II) carbosilane dendrimers bearing aliphatic polyamine ligands presented herein exhibit a rapid and pronounced antitumour reduction achieved with only a single dose in a remarkably short timeframe. Critically, the antitumour effect displayed a clear generation‐dependent profile, with the second‐generation dendrimer **8** inducing the most substantial reduction in tumour burden, exceeding 65%. This level of efficacy is comparable to many nanotherapeutic systems previously evaluated in similar preclinical melanoma models. Taken together, these findings establish a strong foundation for the continued development of ruthenium‐based metallodendrimers. Ongoing and future studies will focus on elucidating their pharmacokinetic behaviour, safety profile and scalability, as well as exploring alternative routes of administration, thereby supporting the progression of these macromolecular platforms toward clinically relevant applications.

## Author Contributions

Yoel Garrosa‐Miró: writing–review and editing, investigation, formal analysis and methodology, Lorena García Hevia: writing–review and editing, resources, methodology, investigation, formal analysis and funding acquisition. Mónica L. Fanarraga: writing–review and editing, resources, funding acquisition and conceptualization. Paula Ortega: writing–original draft, writing–review and editing, methodology, resources, funding acquisition, conceptualization and supervision. Francisco Javier de la Mata: writing–review and editing, methodology, resources, funding acquisition, conceptualization and supervision.

## Funding

This study was supported by Ministerio de Ciencia e Innovación, PID2024‐156624OB‐I00 TED2021‐129248B‐10 RYC2022‐035230‐I; Universidad de Alcalá, PIUAH21/CC‐041 EPU‐INV‐UAH/2021/002; Junta de Comunidades de Castilla‐La Mancha, SBPLY/23/180,225/000,109; and Instituto de Salud Carlos III, FORT23/00,046 PI22/00,030 PI23/00,261 DTS24/000,237 PI25/00,235.

## Conflicts of Interest

The authors declare no conflicts of interest.

## Supporting Information

Additional supporting information can be found online in the Supporting Information section.

## Supporting information


**Supporting Information** Figure S1. ^1^H‐NMR and ^13^C{^1^H}‐NMR spectra of G_1_{[N[(CH_2_)_3_Pht]_2_]_4_} **(1)** in CDCl_3_. Figure S2. ^1^H‐^13^C HSQC and ^1^H‐^1^H COSY spectra of G_1_{[N[(CH_2_)_3_Pht]_2_]_4_} **(1)** in CDCl_3_. Figure S3. ^1^H‐NMR and ^13^C{^1^H}‐NMR spectra of G_2_{[N[(CH_2_)_3_Pht]_2_]_8_} **(2)** in CDCl_3_. Figure S4. ^1^H‐^13^C HSQC and ^1^H‐^1^H COSY spectra of G_2_{[N[(CH_2_)_3_Pht]_2_]_8_}**(2)** in CDCl_3_. Figure S5. ^1^H‐NMR and ^13^C{^1^H}‐NMR spectra of G_1_{[N[(CH_2_)_3_NH_2_]_2_]_4_} **(3)** in CDCl_3_. Figure S6. ^1^H‐^13^C HSQC and ^1^H‐^1^H COSY spectra of G_1_{[N[(CH_2_)_3_NH_2_]_2_]_4_} **(3)** in CDCl_3_. Figure S7. ^1^H‐NMR and ^13^C{^1^H}‐NMR spectra of G_2_{[N[(CH_2_)_3_NH_2_]_2_]_8_} **(4)** in CDCl_3_. Figure S8. ^1^H‐^13^C HSQC and ^1^H‐^1^H COSY spectra of G_2_{[N[(CH_2_)_3_NH_2_]_2_]_8_} **(4)** inCDCl_3_. Figure S9. ^1^H‐NMR and ^13^C{^1^H}‐NMR spectra of G_1_{[N[(CH_2_)_3_NCPh]_2_]_4_} **(5)** in CDCl_3_. Figure S10. ^1^H‐^13^C HSQC and ^1^H‐^1^H COSY spectra of G_1_{[N[(CH_2_)_3_NCPh]_2_]_4_} **(5)** in CDCl_3_. Figure S11. ^1^H‐NMR and ^13^C{^1^H}‐NMR spectra of G_2_{[N[(CH2)_3_NCPh]_2_]_8_} **(6)** in CDCl_3_. Figure S12. ^1^H‐^13^C HSQC and ^1^H‐^1^H COSY spectra of G_2_{[N[(CH2)_3_NCPh]_2_]_8_} **(6)** in CDCl_3_. Figure S13. ^1^H‐NMR and ^13^C{^1^H}‐NMR spectra of G_1_{[N[[(CH_2_)_3_NCPh(*o*‐N)]Ru(*η* ^6^‐p‐cymene)Cl_2_]_2_]_4_} **(7)** in CD_3_OD. Figure S14. ^1^H‐^13^C HSQC and ^1^H‐^1^H COSY spectra of G_1_{[N[[(CH_2_)_3_NCPh(*o*‐N)]Ru(*η* ^6^‐p‐cymene)Cl_2_]_2_]_4_} **(7)** in CD_3_OD. Figure S15. ^1^H‐NMR and ^13^C{^1^H}‐NMR spectra of G_2_{[N[[(CH_2_)_3_NCPh(*o*‐N)]Ru(*η* ^6^‐p‐cymene)Cl_2_]_2_]_8_} **(8)** in CD_3_OD. Figure S16. ^1^H‐^13^C HSQC and ^1^H‐^1^H COSY spectra of G_2_{[N[[(CH_2_)_3_NCPh(*o*‐N)]Ru(*η* ^6^‐p‐cymene)Cl_2_]_2_]_8_} **(8)** in CD_3_OD. Figure S17. MS (ESI‐Q) of G_1_{[N[(CH_2_)3Pht]_2_]_4_} **(1)**. Figure S18. Expanded view and detailed assignment of MS (ESI‐Q) of G_1_{[N[(CH_2_)_3_Pht]_2_]_4_} **(1)**. Figure S19. MS (ESI‐Q) of G_2_{[N[(CH_2_)_3_Pht]_2_]_8_} **(2)**. Figure S20. Expanded view and detailed assignment of MS (ESI‐Q) of G_2_{[N[(CH_2_)_3_Pht]_2_]_8_} **(2)**. Figure S21. MS (ESI‐Q) of G_1_{[N[(CH_2_)_3_NH_2_]_2_]_4_} **(3)**. Figure S22. Expanded view and detailed assignment of MS (ESI‐Q) of G_1_{[N[(CH_2_)_3_NH_2_]_2_]_4_} **(3)**. Figure S23. ^1^H‐NMR spectra recorded from 0 to 24 h hour for G_1_{[N[[(CH_2_)_3_NCPh(*o*‐N)]Ru(*η* ^6^‐p‐cymene)Cl_2_]_2_]_4_} **(7)** in D_2_O (∗).

## Data Availability

The data supporting this article have been included as part of the Supporting Information.
