# Supplementary material for: Ruthenium(II) Carbosilane Dendrimers With Aliphatic Polyamine Ligands for In Vivo Antimelanoma Activity
Source: Bioinorg Chem Appl. 2026 Oct 26;2026:4574339. doi: 10.1155/bca/4574339 (PMC13696996; doi:10.1155/bca/4574339)
Supplement: Supplementary file 1 [file BCA-2026-4574339-s001.docx]

**SUPLEMENTARY MATTERIAL**

**Ruthenium(II) carbosilane dendrimers with Aliphatic Polyamine Ligands for In Vivo Antimelanoma Activity**

Yoel Garrosa-Miró ^[a,b]^, Lorena García Hevia^[c,d,e]^ Mónica L Fanarraga^[c,d]^ , Paula Ortega^[a,b]^* and Francisco Javier de la Mata^[a,b]^ *.

List of the Supplementary information:

Figure S1. ^1^H-NMR and ^13^C{^1^H}-NMR spectra of G_1_{[N[(CH_2_)_3_Pht]_2_]_4_} (1) in CDCl_3_.

Figure S2. ^1^H-^13^C HSQC and ^1^H-^1^H COSY spectra of G_1_{[N[(CH_2_)_3_Pht]_2_]_4_} (1) in CDCl_3_.

Figure S3. ^1^H-NMR and ^13^C{^1^H}-NMR spectra of G_2_{[N[(CH_2_)_3_Pht]_2_]_8_} (2) in CDCl_3_.

Figure S4. ^1^H-^13^C HSQC and ^1^H-^1^H COSY spectra of G_2_{[N[(CH_2_)_3_Pht]_2_]_8_}(2) in CDCl_3_.

Figure S5. ^1^H-NMR and ^13^C{^1^H}-NMR spectra of G_1_{[N[(CH_2_)_3_NH_2_]_2_]_4_} (3) in CDCl_3_.

Figure S6. ^1^H-^13^C HSQC and ^1^H-^1^H COSY spectra of G_1_{[N[(CH_2_)_3_NH_2_]_2_]_4_} (3) in CDCl_3_.

Figure S7. ^1^H-NMR and ^13^C{^1^H}-NMR spectra of G_2_{[N[(CH_2_)_3_NH_2_]_2_]_8_} (4) in CDCl_3_.

Figure S8. ^1^H-^13^C HSQC and ^1^H-^1^H COSY spectra of G_2_{[N[(CH_2_)_3_NH_2_]_2_]_8_} (4) inCDCl_3_.

Figure S9. ^1^H-NMR and ^13^C{^1^H}-NMR spectra of G_1_{[N[(CH_2_)_3_NCPh]_2_]_4_} (5) in CDCl_3_.

Figure S10. ^1^H-^13^C HSQC and ^1^H-^1^H COSY spectra of G_1_{[N[(CH_2_)_3_NCPh]_2_]_4_} (5) in CDCl_3_.

Figure S11. ^1^H-NMR and ^13^C{^1^H}-NMR spectra of G_2_{[N[(CH2)_3_NCPh]_2_]_8_} (6) in CDCl_3_.

Figure S12. ^1^H-^13^C HSQC and ^1^H-^1^H COSY spectra of G_2_{[N[(CH2)_3_NCPh]_2_]_8_} (6) in CDCl_3_.

Figure S13. ^1^H-NMR and ^13^C{^1^H}-NMR spectra of G_1_{[N[[(CH_2_)_3_NCPh(*o*-N)]Ru(η^6^-*p*-cymene)Cl_2_]_2_]_4_} (7) in CD_3_OD.

Figure S14. ^1^H-^13^C HSQC and ^1^H-^1^H COSY spectra of G_1_{[N[[(CH_2_)_3_NCPh(*o*-N)]Ru(η^6^-*p*-cymene)Cl_2_]_2_]_4_} (7) in CD_3_OD.

Figure S15. ^1^H-NMR and ^13^C{^1^H}-NMR spectra of G_2_{[N[[(CH_2_)_3_NCPh(*o*-N)]Ru(η^6^-*p*-cymene)Cl_2_]_2_]_8_} (8) in CD_3_OD.

Figure S16. ^1^H-^13^C HSQC and ^1^H-^1^H COSY spectra of G_2_{[N[[(CH_2_)_3_NCPh(*o*-N)]Ru(η^6^-*p*-cymene)Cl_2_]_2_]_8_} (8) in CD_3_OD.

Figure S17. MS (ESI-Q) of G_1_{[N[(CH_2_)_3_Pht]_2_]_4_} (1).

Figure S18. Expanded view and detailed assignment of MS (ESI-Q) of G_1_{[N[(CH_2_)_3_Pht]_2_]_4_} (1).

Figure S19. MS (ESI-Q) of G_2_{[N[(CH_2_)_3_Pht]_2_]_8_} (2).

Figure S20. Expanded view and detailed assignment of MS (ESI-Q) of G_2_{[N[(CH_2_)_3_Pht]_2_]_8_} (2).

Figure S21. MS (ESI-Q) of G_1_{[N[(CH_2_)_3_NH_2_]_2_]_4_} (3).

Figure S22. Expanded view and detailed assignment of MS (ESI-Q) of G_1_{[N[(CH_2_)_3_NH_2_]_2_]_4_} (3).

Figure S23. ^1^H-NMR spectra recorded from 0 to 24 h hour for G_1_{[N[[(CH_2_)_3_NCPh(*o*-N)]Ru(η^6^-*p*-cymene)Cl_2_]_2_]_4_} (7) in D_2_O (*).

Figure S1. ^1^H-NMR and ^13^C{^1^H}-NMR spectra of G_1_{[N[(CH_2_)_3_Pht]_2_]_4_} (1) in CDCl_3_. * asses to 5% of N‑*bis*‑(phthalimidepropyl)amine. The impurities observed around the 0.1 ppm region of the ^1^H NMR spectrum were assigned as follows: 1, an unidentified structural defect of the dendrimer; 2, high-vacuum grease; and 3, a structural dendrimer defect arising from isomerization.

Figure S2. ^1^H-^13^C HSQC and ^1^H-^1^H COSY spectra of G_1_{[N[(CH_2_)_3_Pht]_2_]_4_} (1) in CDCl_3_.

Figure S3. ^1^H-NMR and ^13^C{^1^H}-NMR spectra of G_2_{[N[(CH_2_)_3_Pht]_2_]_8_}(2) in CDCl_3_. * asses to 4% of N‑*bis*‑(phthalimidepropyl)amine. The impurities observed around the 0.1 ppm region of the ^1^H NMR spectrum were assigned as follows: 2, high-vacuum grease; and 3, a structural dendrimer defect arising from isomerization.

Figure S4. ^1^H-^13^C HSQC and ^1^H-^1^H COSY spectra of G_2_{[N[(CH_2_)_3_Pht]_2_]_8_}(2) in CDCl_3_.

Figure S5. ^1^H-NMR and ^13^C{^1^H}-NMR spectra of G_1_{[N[(CH_2_)_3_NH_2_]_2_]_4_} (3) in CDCl_3_. * asses to unidentified impurities. The impurities observed around 0.1 ppm region of the ^1^H NMR spectrum were assigned as follows: 1, an unidentified structural defect of the dendrimer; 2, high-vacuum grease; and 3, a structural dendrimer defect arising from isomerization.

Figure S6. ^1^H-^13^C HSQC and ^1^H-^1^H COSY spectra of G_1_{[N[(CH_2_)_3_NH_2_]_2_]_4_} (3) in CDCl_3_.

Figure S7. ^1^H-NMR and ^13^C{^1^H}-NMR spectra of G_2_{[N[(CH_2_)_3_NH_2_]_2_]_8_} (4) in CDCl_3_. * asses to unidentified impurities. The impurities observed around 0.1 ppm region of the ^1^H NMR spectrum were assigned as follows: 1, an unidentified structural defect of the dendrimer; 2, high-vacuum grease; and 3, a structural dendrimer defect arising from isomerization.

Figure S8. ^1^H-^13^C HSQC and ^1^H-^1^H COSY spectra of G_2_{[N[(CH_2_)_3_NH_2_]_2_]_8_} (4) in CDCl_3_.

Figure S9. ^1^H-NMR and ^13^C{^1^H}-NMR spectra of G_1_{[N[(CH_2_)_3_NCPh]_2_]_4_} (5) in CDCl_3_. * asses to excess of 2-pyridinecarboxaldehyde less than 1%. The impurities observed around 0.1 ppm region of the ^1^H NMR spectrum were assigned as follows: 2, high-vacuum grease; and 3, a structural dendrimer defect arising from isomerization.

Figure S10. ^1^H-^13^C HSQC and ^1^H-^1^H COSY spectra of G_1_{[N[(CH_2_)_3_NCPh]_2_]_4_} (5) in CDCl_3_.

Figure S11. ^1^H-NMR and ^13^C{^1^H}-NMR spectra of G_2_{[N[(CH2)_3_NCPh]_2_]_8_} (6) in CDCl_3_. * asses to excess of 2-pyridinecarboxaldehyde less than 1%. The impurities observed around 0.1 ppm region of the ^1^H NMR spectrum were assigned as follows: 2, high-vacuum grease; and 3, a structural dendrimer defect arising from isomerization.

Figure S12. ^1^H-^13^C HSQC and ^1^H-^1^H COSY spectra of G_2_{[N[(CH2)_3_NCPh]_2_]_8_} (6) in CDCl_3_.

Figure S13. ^1^H-NMR and ^13^C{^1^H}-NMR spectra of G_1_{[N[[(CH_2_)_3_NCPh(*o*-N)]Ru(η^6^-*p*-cymene)Cl_2_]_2_]_4_} (7) in CD_3_OD. * asses to 4% excess of RuCl_2_(η^6^‑p‑cymene)]_2_. The impurities observed around 0.1 ppm region of the ^1^H NMR spectrum were assigned as follows: 2, high-vacuum grease; and 3, a structural dendrimer defect arising from isomerization.

Figure S14. ^1^H-^13^C HSQC and ^1^H-^1^H COSY spectra of G_1_{[N[[(CH_2_)_3_NCPh(*o*-N)]Ru(η^6^-*p*-cymene)Cl_2_]_2_]_4_} (7) in CD_3_OD.

Figure S15. ^1^H-NMR and ^13^C{^1^H}-NMR spectra of G_2_{[N[[(CH_2_)_3_NCPh(*o*-N)]Ru(η^6^-*p*-cymene)Cl_2_]_2_]_8_} (8) in CD_3_OD. * asses to 2% excess of RuCl_2_(η^6^‑p‑cymene)]_2_.

Figure S16. ^1^H-^13^C HSQC and ^1^H-^1^H COSY spectra of G_2_{[N[[(CH_2_)_3_NCPh(*o*-N)]Ru(η^6^-*p*-cymene)Cl_2_]_2_]_8_} (8) in CD_3_OD.

Figure S17. MS (ESI-Q) of G_1_{[N[(CH_2_)_3_Pht]_2_]_4_} (1).

Figure S18. Expanded view and detailed assignment of (ESI-Q) of G_1_{[N[(CH_2_)_3_Pht]_2_]_4_} (1). A) Proposed fragmentation pathways and reactions: a, Hofmann Elimination; b) Iodide addition. B) Table of the main assigned peaks.

Figure S19. MS (ESI-Q) of G_2_{[N[(CH_2_)_3_Pht]_2_]_8_} (2).

Figure S20. Expanded view and detailed assignment of (ESI-Q) of G_2_{[N[(CH_2_)_3_Pht]_2_]_8_} (2). A) Proposed fragmentation pathways and reactions: a, Hofmann Elimination; b) Iodide addition. B) Structural dendrimer defects by isomerization. C) Table of the main assigned peaks.

Figure S21. MS (ESI-Q) of G_1_{[N[(CH_2_)_3_NH_2_]_2_]_4_} (3).

Figure S22. Expanded view and detailed assignment of (ESI-Q) of G_1_{[N[(CH_2_)_3_NH_2_]_2_]_4_} (3). A) Proposed fragmentation pathways and reactions: a, Hoffmann Elimination. B) Table of the main assigned peaks.

Figure S23. ^1^H-NMR spectra recorded from 0 to 24 h hour for G_1_{[N[[(CH_2_)_3_NCPh(*o*-N)]Ru(η^6^-*p*-cymene)Cl_2_]_2_]_4_} (7) in D_2_O (*).
